# The Importance of Ultrasound-Guided Synovial Biopsy in the Workup of Seronegative Inflammatory Arthritis: A Case Report

**DOI:** 10.7759/cureus.53805

**Published:** 2024-02-07

**Authors:** Cherie S Zhi, Julia R Kesselhaut, Swamy R Venuturupalli, Ami Ben-Artzi

**Affiliations:** 1 Rheumatology, Cedars-Sinai Medical Center, Los Angeles, USA

**Keywords:** hand and wrist pain, congo red, synovial tissue, seronegative rheumatoid arthritis, inflammatory arthritis, transthyretin cardiac amyloidosis, ultrasound-guided synovial biopsy

## Abstract

We report a case of a 74-year-old male who presented with typical clinical features of rheumatoid arthritis (RA), as well as elevated markers of inflammation. However, the patient did not respond to multiple RA treatments, and an ultrasound-guided synovial biopsy (UGSB) of the right wrist was performed, which established the diagnosis of amyloidosis. A variety of inflammatory conditions sometimes get misdiagnosed as seronegative RA due to similarities in clinical presentation. This case report highlights the importance of a thorough workup in patients who appear to have seronegative RA. Given the wide availability of ultrasound-guided, minimally invasive synovial biopsies, these procedures should be employed more often to detect rare conditions that may mimic seronegative RA, such as amyloidosis.

## Introduction

Rheumatoid arthritis (RA) is an autoimmune disease characterized by a variety of clinical presentations of joint inflammation, as well as extra-articular manifestations, such as interstitial lung disease [1]. It is caused by a dysregulation of the immune system, which recognizes proteins as antigens rather than normal proteins. This promotes cellular immune responses in the form of inflammation, bone erosion, and cartilage degradation. The diagnosis of RA is made based on both clinical features and laboratory tests. The 2010 American College of Rheumatology (ACR)/European League Against Rheumatism (EULAR) RA classification criteria involve a scoring system for RA diagnosis, comprising the following four categories: joint involvement, serology [rheumatoid factor (RF)/anti-cyclic citrullinated peptide (CCP)], acute phase reactants [erythrocyte sedimentation rate (ESR)/C-reactive protein(CRP)], and duration of symptoms (more than six weeks) [2]. Seronegative RA is diagnosed in patients who do not have positive RF/CCP [1]. A variety of inflammatory conditions sometimes get misdiagnosed as seronegative RA due to the similarity in clinical presentation, raising the possibility that a segment of patients diagnosed with seronegative RA is misclassified [1].

Amyloidosis is caused by a misfolding of amyloid proteins, leading to the aggregation of insoluble amyloid fibrils around organs and other parts of the body [3]. Cardiac transthyretin (TTR) amyloidosis involves the deposition of TTR protein, which carries thyroxine and retinol-binding protein to the body [4]. The diagnosis of amyloidosis is based on the presence of amyloid protein in a biopsy of the affected organ, which is detected using Congo Red, a thioflavin dye [5]. TTR amyloidosis has been reported to be associated with carpal tunnel syndrome [4]. However, it is less common to have TTR amyloidosis causing joint inflammation and elevated acute phase reactants, thereby mimicking the clinical presentation of seronegative RA [6].

## Case presentation

**Figure 1:**
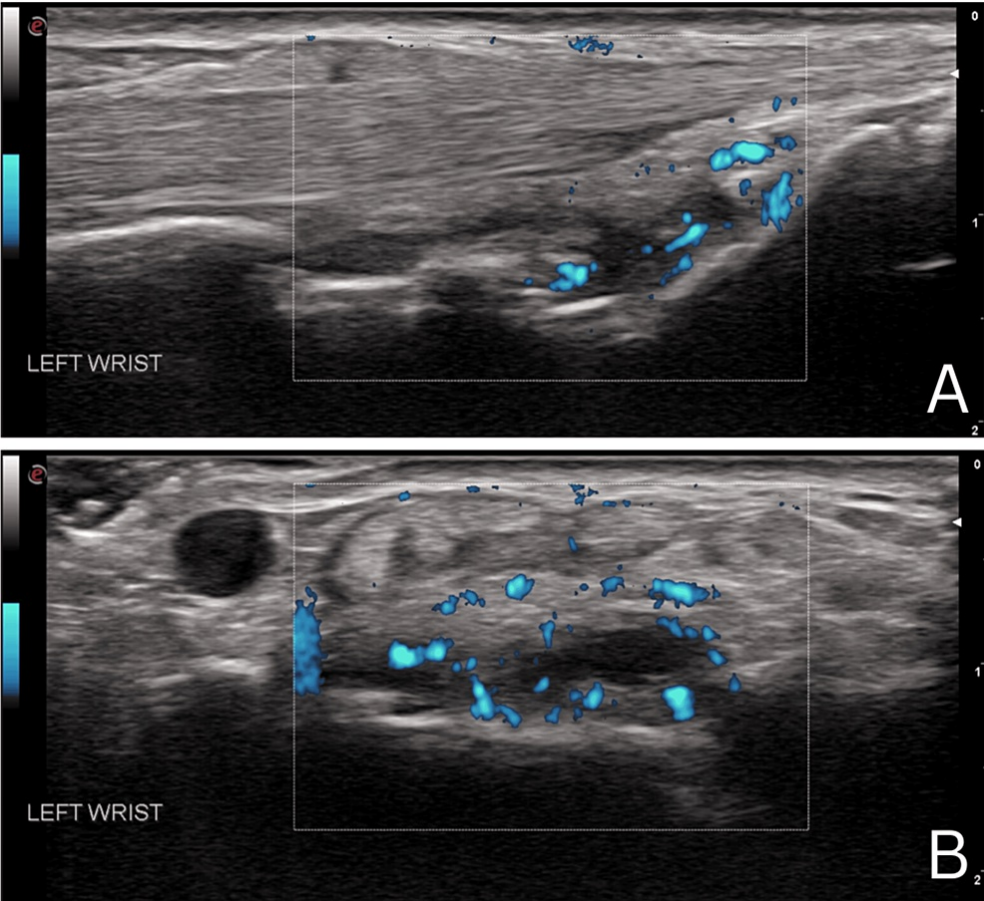
Ultrasound exam of the left dorsal wrist showing Doppler-positive synovitis in the midline long axis view (A) and short axis view (B)

A 74-year-old male presented with symptoms of bilateral hand pain and numbness, as well as wrist pain. He had undergone a bilateral carpal tunnel release surgery, after which his symptoms had somewhat improved. However, limited range of motion and pain in the wrists had persisted, which had led the patient to pursue a rheumatology evaluation. A diagnostic ultrasound exam of the right wrist found moderate synovitis with a moderate Doppler signal, suggestive of active inflammatory disease (Figures 1A-1B). His CRP level was found to be elevated at 23 mg/L, although RF and CCP antibodies were negative.

**Figure 2:**
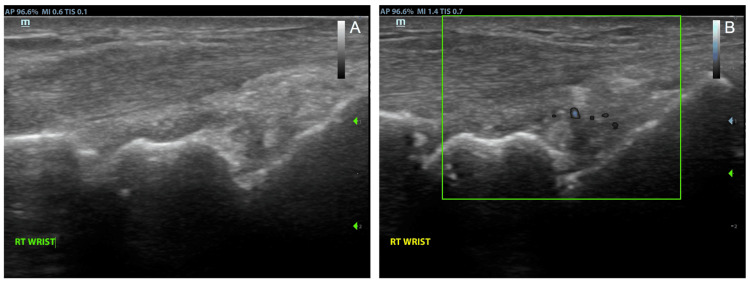
Repeat ultrasound exam of the wrist in dorsal midline axis view in grayscale (A) and with power Doppler signal (B) showing persistent inflammation after failing three types of RA treatments RA: rheumatoid arthritis

Despite not meeting the criteria for a diagnosis of RA, due to his features suggestive of seronegative RA, the patient was offered a trial of RA pharmaceutical therapies with the possibility of improved clinical outcomes. He tried methotrexate with certolizumab pegol for five months, and then tocilizumab for five months, but had no subjective improvement in symptoms. One year after the patient’s initial evaluation, an ultrasound exam of the right wrist showed that mild to moderate synovitis was still present, although with a trace Doppler signal (Figures 2A-2B). The patient’s CRP level was still elevated, at 39.8 mg/L.

**Figure 3:**
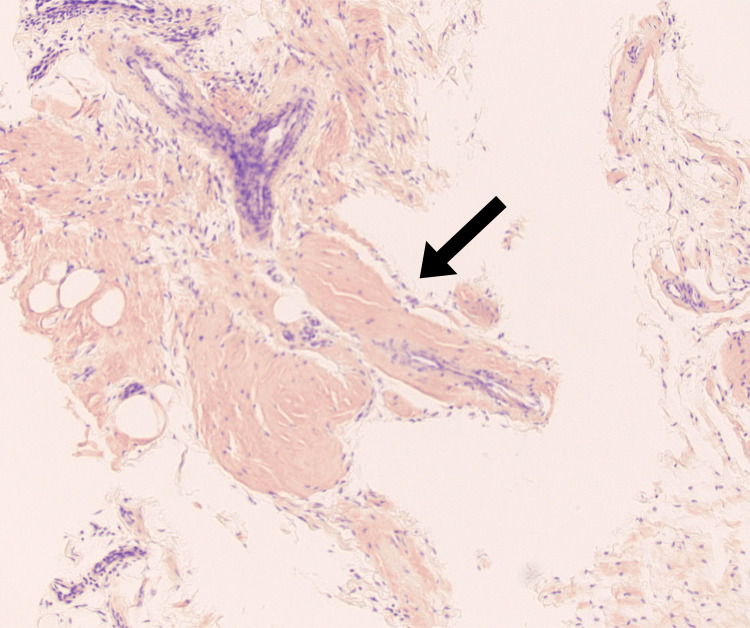
Histologic exam of synovial tissue Congo Red stain was applied to the tissue, and the bright orange color indicates the presence of amyloid deposits

The patient also presented with mild hypertension, and given his history of carpal tunnel syndrome symptoms, the possibility of cardiac TTR amyloidosis was raised by his cardiologist. To further investigate the patient’s seronegative RA symptoms, an ultrasound-guided synovial biopsy (UGSB) of the left wrist was performed. Upon pathological analysis, synovial tissue was found to have depositions identified as amyloid (Figure 3). The histologic exam was positive for Congo Red, confirming amyloid deposition.

## Discussion

In many cases, patients who lack RF/CCP antibodies but manifest other symptoms of RA as well as elevated acute phase reactants may be diagnosed with seronegative RA. This raises the possibility that a minority of patients who have been diagnosed with RA may actually have other underlying diseases mimicking a rheumatologic condition. Our patient presented with bilateral hand and wrist pain with synovitis, elevated CRP, and symptoms lasting for more than three years, which were suggestive of seronegative RA, but ultimately did not meet the criteria for RA diagnosis. After failing three types of RA treatments, further workup via UGSB identified amyloidosis to be affecting the wrist. As demonstrated in this case, it is important to conduct a thorough workup for patients with features of seronegative RA.

A review of the literature showed that carpal tunnel syndrome is a common early clinical feature of cardiac TTR amyloidosis, but few reports describe synovitis and elevated CRP associated with TTR amyloidosis [4,6,7]. The finding of moderate synovitis with moderate Doppler signal in the wrist, which is strongly associated with active inflammatory disease, is an uncommon feature of cardiac TTR amyloidosis. However, based on the identification of amyloid deposits, this finding highlights the utility of UGSB in reaching an accurate diagnosis explaining persistent synovitis.

The safety and wide availability of minimally invasive UGSB make it a valuable tool in the diagnosis of various conditions including infection, crystal arthritis, autoimmune arthritis, and malignancy [8]. Synovial biopsies are generally safe, well-tolerated, and inexpensive [8]. Compared to obtaining synovial tissue via arthroscopy, UGSB is less invasive as it is performed as a bedside procedure [8]. It can be used to access large, medium, and small joints, which is beneficial in the workup of patients with seronegative RA symptoms [8]. Compared to blind-needle and fluoroscopy-guided biopsies, UGSB is more precise and has a higher success rate [9]. Overall, the precision and safety of UGSB should make it a first-line diagnostic tool in patients with features of seronegative RA.

## Conclusions

Seronegative inflammatory arthritis should be further worked up with UGSB of the affected joint. The use of UGSB was critical in accurately diagnosing our patient’s inflammatory arthritis symptoms and establishing the diagnosis of cardiac TTR amyloidosis.
